# Multimodal environmental cleaning strategies to prevent healthcare-associated infections

**DOI:** 10.1186/s13756-023-01274-4

**Published:** 2023-08-23

**Authors:** Katrina Browne, Brett G Mitchell

**Affiliations:** 1 School of Nursing and Health, Avondale University, Cooranbong, NSW Australia; 2 Central Coast Local Health District, Gosford Hospital, Gosford, NSW Australia; 3 School of Nursing and Midwifery, Monash University, Melbourne, VIC Australia; 4 Hunter Medical Research Institute, Newcastle, NSW Australia

**Keywords:** Healthcare-associated infection, Infection prevention, Infection control, Cleaning and disinfection, Cost effectiveness, Cross-infection, Health services

## Abstract

Infection transmission in healthcare is multifaceted and by in large involves the complex interplay between a pathogen, a host and their environment. To prevent transmission, infection prevention strategies must also consider these complexities and incorporate targeted interventions aimed at all possible transmission pathways. One strategy to prevent and control infection is environmental cleaning. There are many aspects to an environmental cleaning strategy. We believe the key to successfully reducing the risk of healthcare-associated infections through the environment, is to design and implement a multimodal intervention. This paper aims to provide an overview of important considerations for designing a meaningful and sustainable environmental program for healthcare facilities.

## Introduction

Healthcare-associated infections (HAIs) are a frequent and preventable adverse event resulting from medical care [1, 2]. HAIs impose an enormous financial burden due to prolonged hospital stays and ongoing treatment costs, as well as increased morbidity and mortality [3, 4]. Pathogens may be transmitted from the environment (exogenous) or from the patient’s own microbiota (endogenous). Environmental hygiene initiates are concerned with preventing exogenous transmission of pathogens in hospitals. Pathogens can persist in the healthcare environment after cleaning, where they remain viable [5]. Some of the strongest epidemiological evidence of environmental transmission is shown through the increased risk of infection if a prior room occupant was infected [6]. Findings from randomized controlled trials have also demonstrated that it is possible to reduce HAIs through changes and improvements to environmental cleaning [7, 8]. There are also many examples where cleaning interventions have been used to reduce infection transmission in outbreaks scenarios, as well as in non-randomised trials [9, 10].

Infection transmission is multifaceted and generally involves the complex interplay between a pathogen, a host and their environment (including humans) – requiring multifaceted strategies to prevent their transmission. There are also important contextual factors that are likely to play a role in the effectiveness of infection prevention strategies, including organizational culture, governance, support, resources, risk appetite and motivation and capacity to change [9]. There have been notable examples of the value of multimodal approaches in other areas of infection prevention, including the Michigan project and Five Moments of Hand Hygiene initiative [11, 12]. For example, multimodal hand hygiene strategies include a system change, training and education, institutional security climate, reminders, evaluation and feedback and administrative support [13]. Multimodal strategies increase the effectiveness and sustainability of initiatives [14–16]. However, one challenge with ‘bundled’ or multimodal approaches to infection prevention is determining the relative benefit of each component of any given bundle [17]. This may be important where limited resources are available restricting the ability to implement all components. Infection prevention recommendations, including cleaning recommendations, are largely underpinned by quasi-experimental and observational based studies.

There has been a rapid increase in the number of published articles on the topic of environmental cleaning in healthcare facilities over the past 20 years, from less than 100 papers in 2008, to over 1000 in 2022 (data sourced from Google Scholar). A systematic review examining the effect of healthcare environmental hygiene interventions on HAIs, identified eight studies published up the year 2020, that used a bundled (or multimodal) approach [9]. Of these eight studies, six were published since 2016 [7, 8, 18–21].

Using evidence from these multimodal studies, and those conducted in other areas of infection prevention, we present important considerations when designing multimodal environmental cleaning strategies to prevent healthcare-associated infections. The focus of this review is hospital settings and cleaning approaches primarily delivered by cleaning or environmental services staff; however, the general principles are applicable to the wider healthcare settings. For example, aged care facilities, imaging centers, general practice and office-based practice may have different needs and therefore different environmental cleaning approaches. In all settings, it is important to conduct a thorough risk assessment prior to implementing environmental cleaning initiatives.

## Environmental cleaning strategies to reduce the risk of healthcare-associated infections

**Fig. 1:**
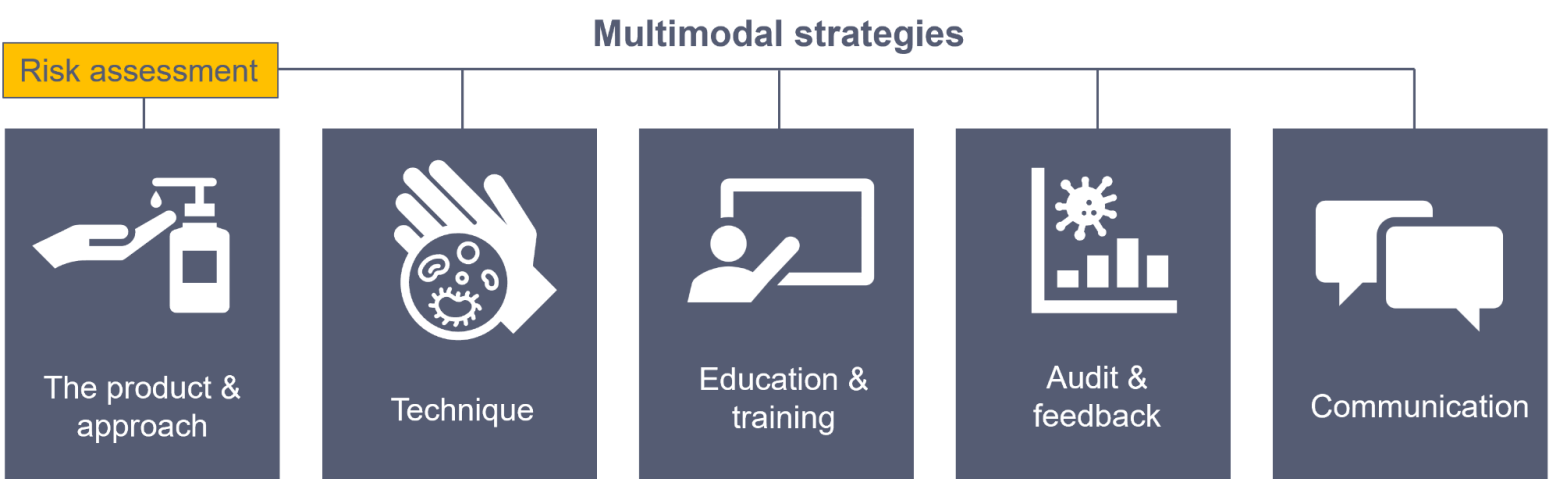
A multimodal approach to environmental cleaning in healthcare facilities encompasses five key strategies: the product and approach used for cleaning, technique, education and training, audit and feedback, and communication (adapted from REACH study [7]).

There are several important inter-related environmental cleaning strategies that are used to reduce the risk of HAIs. These are summarized in Fig. 1 and include, the product and approach used for cleaning, technique, education and training, audit and feedback, and communication. These are based on the bundled approach of the REACH (Researching Effective Approaches to Cleaning in Hospitals) trial [7]. Importantly, these approaches must begin with a risk assessment prior to implementing an environmental cleaning initiative [22]. We will explore each of the strategies in more detail, in this section, drawing on the wider literature.

### The product and approach to cleaning

In deciding the approach to environmental cleaning, it is important to consider the risks relative to your institution and patients. Three important considerations include the patient risk profile, surface risk profile and the pathogen risk profile [22]. The patient risk profile refers to the vulnerability of patients or clients. The surface risk profile is the likelihood of contamination with pathogens and the risk for further transmission. The pathogen risk profile category refers to the persistence of viable pathogens, antimicrobial resistance considerations and main modes of pathogen transmission [22]. Further detail on a risk-based approach to cleaning is detailed in a review by Assadian and colleagues [22].

Cleaning is comprised of the physical removal of dirt, oils and debris on a given surface, usually with a soap or detergent cloth/wipe. Disinfection is an enhanced cleaning method which aims to eliminate or reduce harmful pathogens on surface and is most effective when pathogens have been physically removed from a surface using a cleaning process [23]. Disinfectants may not work effectively in the presence of residual surface soil [24]. Disinfection may occur following a cleaning procedure or can be undertaken in conjunction with cleaning in some instances. A large variety of disinfectants and disinfection processes are available for use, including alcohols, aldehydes, amines, chlorines, oxidative agents (e.g., hydrogen peroxide and peracetic acid), phenols, quaternary ammonium compounds and ultraviolet-C (UV-C). Each have their own characteristics and limitations [22]. The decision to use disinfection measures as part of a cleaning intervention should be informed by a risk assessment, as summarized earlier. Various resources are available to help determine clinical risk and appropriate choice of cleaning and disinfection methods [22, 25, 26]. Compatibility of disinfectants and materials should be assessed prior to disinfection.

Cleaning and/or disinfection may involve the use of cloths, wipes, mops, buckets and sponges. Similarly, it may include “no-touch” devices such as UV-C or hydrogen peroxide vapor disinfection [27, 28]. Only approved products (by the relevant regulatory authority) should be used. Following manufacturer’s instructions for any product is critical. There are several factors to consider when choosing between the different approaches used for cleaning and or disinfection. Some important considerations are summarized in Table 1, noting that depending on the cleaning approach used, not all of these are relevant.

### Technique

The technique used for cleaning is vitally important and has been well described by Dancer and colleagues [29]. The process of cleaning described by Dancer, includes four critical steps – Look, Plan, Clean and Dry. The “Look” step includes describes the need for a a visual assessment of the area to be cleaned. The “Plan” step outlines why an area needs to be prepared before cleaning. The third step, “Clean”, describes the process of cleaning. The final step, “Dry” summarises the need for surfaces to be allowed to dry [29].

**Table 1:** Considerations when choosing a cleaning product or approach to cleaning

Topic	Further detail
Health and safety	Health and safety considerations may include ergonomics and any risk(s) associated with preparation or implementation of cleaning and disinfection [22]. Appropriate personal protective equipment should be worn when using chemicals for cleaning and disinfection [30]
Preparation	Include time to prepare relevant solutions and the area for disinfection. Solutions should be prepared fresh daily, or sooner, according to manufacturer’s instructions [31]. Mop water should be discarded and replaced with fresh detergent solution between rooms (including bathrooms), or every 15 min [29]. Ensure there are enough supplies available for the duration of cleaning. For example, in high-risk areas, cloths must be changed between each patient zone [32].
Contact time	Ensure the products wet-contact time (the time that a disinfectant needs to stay wet on a surface to ensure efficacy) follows manufacturer’s instructions [24]. This may require multiple applications to achieve sufficient contact time [33].
Reprocessing	Cleaning cloths must be changed between patient rooms and bathrooms to avoid cross contamination [29]. Mop heads should be single use or removable for daily laundering and changed between rooms (including bathrooms) [34]. Colour-coded equipment can be used to differentiate between cleaning zones [35].
Storage	Cleaning cloths and mop heads should be laundered with detergent after use. When materials are completely dry, they should be stored in a sealed container [29]. Mop buckets should be stored upside down on a suitable surface to allow drainage [35].
Compatibility	Ensure the cleaning product is compatible with the material/equipment to be cleaned [36]. Consider how the product may interact with any monitoring approach e.g., microbiological sampling and ATP.
Efficacy	The efficacy of the product includes the spectrum of activity and sporicidal activity if needed [37]. Review and consider the supporting evidence.
Transferability of pathogens	Transferability includes the ability to transfer pathogens from one surface to another as part of the cleaning procedure [38].
Practical considerations	A cleaning schedule should be developed outlining equipment to be cleaned, frequency of cleaning and responsible persons [26].

The process of cleaning (“Clean” step) involves several important components. These include the direction of cleaning and the wiping action. The principles involved in the direction of cleaning include cleaning from high to low, clean sites nearest to the patient first, priorisiting hand-touch and frequent touched sites, and cleaning sites from least visually dirty to obviously dirty [29, 39, 40]. The processing wiping a surface involves using one wipe/cloth for each site, unfolding the wipe/cloth and using it flat on the surface, wiping in the one direction and using and S-shaped pattern when wiping [29]. The principle of the ‘one wipe; one site; one direction’ is an useful way to help remember important concepts [29, 41, 42].

It is important to note that cloths need to be changed between rooms (including between patient rooms and bathrooms) as well as within patient rooms for different hand-touch sites – to avoid cross-contamination. For this reason, many hospitals use single-use pre-impregnanted detergent and/or disinfectant wipes. Another important consideration is the “wet contact time” of a disinfectant, and to ensure enough product is used to meet the manufacturers instructions. This may require mulitple applications to meet the required wet contact time, or a specialised product to disinfect persistent pathogens (e.g. *Clostridioides difficile* spores) [24].

### Education and training

A successful intervention or initiative requires education and training of those involved. In the context of environmental cleaning, core education should include content on cleaning roles and responsibilities and the effect of environmental cleaning on reducing healthcare-associated infections. Training should include the cleaning technique and sequence, correct product use and and adherence to manufacturers’ instructions as described above [7].

Training and the educational approach used should be tailored to meet local needs and reflect the context of the respective healthcare setting, including current cleaning approaches (and products), as well as the cleaning schedules [7, 43]. Understanding baseline levels of knowledge and attitude of environmental services staff also allows for the tailoring of training, and may be useful in monitoring future changes [43]. It should be noted that healthcare facilities have multicultural staff, with different language backgrounds. Staff training should incorporate a variety of non-language based strategies, such as visual and kinaesthetic. For example, colour-coding of cloths, mop heads and buckets can help differentiate between equipment for different purposes.

Evaluation of education and training, through feedback from participants, is also important, so that future amendments can be made as required. Ongoing education and training are important, for example through ‘refresher’ training session for ongoing sustainbilty and success [44]. Refresher training sessions.

### Audit and feedback

The use of audit and feedback to drive and sustain improvements in infection control practices, including cleaning, has been well documented in many studies [12, 45, 46]. In the context of environmental cleaning, the use of fluorescent technology to identify the thoroughness of cleaning and provide feedback to environmental services staff is common and is shown to be effective at improving cleaning, as well as reducing HAIs [7, 47, 48]. Fluorescent technology uses invisible gel, paint or powder applied to surface, that are easily removed during normal cleaning processes [49]. A UV light is shone over the equipment to determine the thoroughness of cleaning (if the fluorescent mark has been removed). Different products have varying visibility on surfaces, care should be taken to practise the correct application technique to reduce visible residue on surfaces.

Feedback includes sharing audit results with staff involved, the department, and more broadly with the hospital, including to the appropriate governance committee. Feedback should be timely, individualized, non-punitive and customizable [50]. Audit and feedback may be most effective when it also includes the person responsible for feedback being a supervisor or manger, it is provided more than once, it is delivered verbally and in writing and includes targets and or an action plan [51].

There are other ways in which environmental cleaning and cleanliness can be assessed, including the use of Adenosine Triphosphate (ATP) bioluminescence assays, visual inspections and microbial cultures. All of these processes have advantages and disadvantages, and should be tailored to the specific needs of the facility (for example, outbreak situations may require environmental sampling to detect specific pathogens) [52].

### Communication

Communication is a critical component of commencing and sustaining facility wide infection control initiatives. Environmental services staff are often an ‘invisible workforce’ that are the lowest paid for the service they provide and the importance of their role in the wider context of patient safety [52]. Communication strategies are key to engage staff, support an organizational culture shift and raise the profile of cleaning and environmental services staff. Strategies to achieve this include recognition and reward schemes, facilitating daily contact between cleaning staff and clinical staff, as well as reports to appropriate governance committees, leaders and managers [7, 12].

Visual reminders, may also be a useful form of communication [53]. Reminders include those that target environmental services staff, by providing helpful information relevant to key aspects of this job, for example the technique of cleaning or product preparation. Visual cues to the wider organization about the important work environmental cleaning staff do may also be of benefit to morale.

## Discussion

In this paper, we have outlined a framework for a multimodal approach to environmental cleaning. When describing these individual aspects, we have provided a high-level overview. For additional information, we encourage readers to use the supporting references for further detail. The context of the individual organization, and people, must be considered prior to planning and implementing environmental cleaning initiatives. It is essential to these initiatives are subject to a risk-based assessment and incorporate the five key strategies proposed in this paper. Throughout different healthcare facilities, there is considerable variation in products used, frequency of cleaning, baseline and ongoing training received, staffing models for environmental services staff, ability and readiness to accept change and organizational culture across healthcare facilities [29, 54, 55]. Understanding these and using an implementation framework to guide changes in cleaning practices may be useful [55]. While the framework proposed is most appropriate for cleaners and cleaning services, there are many other situations where cleaning is required and undertaken, for example by clinicians cleaning equipment between patients. There may be elements of the proposed framework that may still be relevant, however there is a need for further research in this area.

Another important challenge in implementing any infection control initiative is cost. Evidence from the multi-centred REACH study demonstrated that implementing a cleaning bundle, consistent with what is proposed in this paper does generate some additional costs, but also results in cost savings. Following a cost-effectiveness evaluation of this study, there was an 86% chance that implementing the cleaning bundle was cost effective, compared with existing hospital cleaning practices [56]. This resulted in an incremental cost-effectiveness ratio of $4684 per quality adjust life year gained [56]. For hospitals in particular, a reduction in HAI rates also reduces patient bed days and antibiotic use [56].

The success of environmental cleaning initiatives is intricately linked to hand hygiene and air quality considerations. There are important synergies between hands and the environment, as well as air. Hands become contaminated when touching surfaces and likewise, contaminated hands have the potential to transfer pathogens to surfaces [57]. A comprehensive hand hygiene program that results in high hand hygiene compliance is therefore theoretically likely to have some impact on the transfer of pathogens from the environment – albeit this is very difficult to quantify [58, 59]. Like hands, there are interactions between the air and surfaces. Infectious pathogens in the air, produced through aerosol producing procedures and or behaviours have the potential to settle surfaces and pose an ongoing risk of transmission [60]. Improving air quality is an important consideration in the broader context of reducing contamination on surfaces.

Cleaning and disinfection in healthcare facilities is crucial for the prevention of healthcare-associated infections. A robust training and assessment program is needed for healthcare workers to ascertain the underlying microbiology and disease transmission from contaminated surfaces. Enhanced training for these specialized tasks would necessitate enhanced renumeration for the important work they do, and thus help retain high-quality staff.

## Conclusions

For a sustainable infection prevention program, a multimodal strategic approach covers the multifaceted disease transmission pathways in healthcare facilities.

## Data Availability

Data sharing is not applicable to this article as no datasets were generated or analysed during the current study.

## List of abbreviations

ATP: Adenosine Triphosphate
HAI: Healthcare-associated infection
REACH: Researching Effective Approaches to Cleaning in Hospitals
UV-C: Ultraviolet-C
